# Prevalence of diabetes mellitus in Brazil: a systematic review with meta-analysis

**DOI:** 10.1186/1758-5996-7-S1-A195

**Published:** 2015-11-11

**Authors:** Felipe Vogt Cureau, Gabriela Heiden Teló, Martina Schaan de Souza, Fabiana Silveira Côpes, Beatriz D'Agord Schaan

**Affiliations:** 1 UFRGS-Universidade Federal do Rio Grande do Sul, Porto Alegre, Brazil

## Background

The prevalence of diabetes mellitus (DM) is increasing worldwide. The global prevalence is estimated to increase by about 2.2% per year; however, no data is available to evaluate the trends in Brazil over time.

## Aims

We developed a systematic review with meta-analysis to estimate the prevalence and trends of DM in Brazilian adults.

## Materials and methods

Cross-sectional and cohort studies published between 1980 and 2014 were independently identified by two reviewers, without language restriction, in five databases (PubMed, Cochrane Library, EMBASE, LILACS and SciELO). Random effects models were used to estimate the prevalence of DM for the general population, as well as the trends for the last decades. Heterogeneity was assessed by I^2^ statistics.

## Results

In total, 47 articles were selected and included in this review. Three different patterns for the DM diagnosis were identified: self-report (33 studies), fasting plasma glucose (7 studies), and complex diagnostic (e.g. fasting glucose + OGTT + self-report; 7 studies). A meta-analysis was conducted according to the diagnosis pattern. The prevalence of DM was 11.9% (CI95% 7.7-17.8; I^2^=100%) by complex diagnosis, 6.6% (CI95% 4.8-8.9; I^2^=94%) by fasting glucose, and 5.5% (CI95% from 4.9 to 6.2; I^2^=99%) by self-report. In trend analysis, we observed an increase in the prevalence of DM in studies using a complex diagnostic: 7.4% (CI95% 7.1-7.7) in the 1980's, 12.1% (CI95% 10.5-13.8) in the 1990's, 14.5% (CI95% 13.1-16.0) in the 2000's, and 15.7% (CI95% 9.8-24.3) in the 2010's. Although with a lower prevalence, similar trends were observed by self-reported diagnosis: 3.2% (CI95% 2.6-4.1) in the 1990's, 5.7% (CI95% 5.1-6.4) in the 2000's, and 6.8% (CI95% 5.9-7.9) in the 2010's. Only one study evaluated the prevalence of diabetes by fasting glucose in the 1990's (10.3% [CI95% 9.1-11.6]); the other studies were conducted in the 2000's (6.0% [CI95% 4.2-8.6]).

## Conclusions

We identified three methods used to access the prevalence of DM in epidemiological studies in Brazil. Despite the high heterogeneity, studies based on a complex diagnosis showed a high prevalence of DM in Brazilian adults over time (11.9%), with a progressive increase in the last 35 yrs. This trend was also observed in studies based on self-reported diagnosis; however, these findings may be associated with improvement in access to health services in the same period.

